# Enumeration and phenotypical analysis of distinct dendritic cell subsets in psoriatic arthritis and rheumatoid arthritis

**DOI:** 10.1186/ar1864

**Published:** 2005-12-16

**Authors:** Sarah L Jongbloed, M Cristina Lebre, Alasdair R Fraser, J Alastair Gracie, Roger D Sturrock, Paul P Tak, Iain B McInnes

**Affiliations:** 1 Division of Immunology, Infection and Inflammation, 10 Alexandra Parade, Glasgow, G31 2ER, UK; 2 Director, Academic Medical Center, Division of Clinical Immunology and Rheumatology, Academic Medical Center/University of Amsterdam, F4-218 P.O. Box 22700, 1100 DE Amsterdam, The Netherlands

## Abstract

Dendritic cells (DCs) comprise heterogeneous subsets of professional antigen-presenting cells, linking innate and adaptive immunity. Analysis of DC subsets has been hampered by a lack of specific DC markers and reliable quantitation assays. We characterised the immunophenotype and functional characteristics of psoriatic arthritis (PsA)-derived and rheumatoid arthritis (RA)-derived myeloid DCs (mDCs) and plasmacytoid DCs (pDCs) to evaluate their potential role in arthritis. Circulating peripheral blood (PB) pDC numbers were significantly reduced in PsA patients (*P *= 0.0098) and RA patients (*P *= 0.0194), and mDCs were significantly reduced in RA patients (*P *= 0.0086) compared with healthy controls. The number of circulating mDCs in RA PB was significantly inversely correlated to C-reactive protein (*P *= 0.021). The phenotype of both DC subsets in PsA PB and RA PB was immature as compared with healthy controls. Moreover, CD62L expression was significantly decreased on both mDCs (PsA, *P *= 0.0122; RA, *P *= 0.0371) and pDCs (PsA, *P *= 0.0373; RA, *P *= 0.0367) in PB. Both mDCs and pDCs were present in PsA synovial fluid (SF) and RA SF, with the mDC:pDC ratio significantly exceeding that in matched PB (PsA SF, *P *= 0.0453; RA SF, *P *= 0.0082). pDCs isolated from RA SF and PsA SF displayed an immature phenotype comparable with PB pDCs. RA and PsA SF mDCs, however, displayed a more mature phenotype (increased expression of CD80, CD83 and CD86) compared with PB mDCs. Functional analysis revealed that both SF DC subsets matured following toll-like receptor stimulation. pDCs from PB and SF produced interferon alpha and tumour necrosis factor alpha on TLR9 stimulation, but only SF pDCs produced IL-10. Similarly, mDCs from PB and SF produced similar tumour necrosis factor alpha levels to TLR2 agonism, whereas SF mDCs produced more IL-10 than PB controls. Circulating DC subset numbers are reduced in RA PB and PsA PB with reduced CD62L expression. Maturation is incomplete in the inflamed synovial compartment. Immature DCs in SF may contribute to the perpetuation of inflammation via sampling of the inflamed synovial environment, and *in situ *presentation of arthritogenic antigen.

## Introduction

The inflammatory arthritides, including rheumatoid arthritis (RA) and psoriatic arthritis (PsA), comprise autoimmune disorders characterised by chronic joint inflammation, immune cell infiltration to the synovium, fibroblast-like synoviocyte expansion and destruction of cartilage and bone. Extensive *in vivo *and *in vitro *studies have identified multiple proinflammatory cytokines and enzymes implicated in the pathogenesis of both of these disorders. Targeting cytokines has proven therapeutically useful, exemplified particularly in tumour necrosis factor (TNF) blockade and more recently in targeting IL-6 and IL-15 [1-4]. Cytokine blockade, however, exhibits variable responses across patient populations – and, importantly, disease activity recurs upon cessation of therapy. A major challenge in therapeutics now is to develop strategies that re-establish immune tolerance such that amelioration of inflammation is accompanied by long-term disease suppression. Early evidence that such responses may be feasible is derived from clinical studies utilising CTLA-4Ig [5] and anti-CD20 [6,7] in RA, and using co-stimulatory blockade in psoriasis [8-10].

Many data indicate the presence and significance of autoimmune processes in articular inflammation. Recent results in RA patients implicate citrullinated peptides in disease progression and response to TNF blockade [11]. Additional auto-specific antigens include type II collagen [12] and cartilage-derived gp39 [13,14]. Although few autoimmune targets have been identified in PsA, perhaps reflecting the absence thus far of defined autoantibodies, analysis of PsA synovial and skin T cell subsets clearly indicates oligoclonal responses that may be self-targeted [15,16]. Critically, in both diseases, upstream immunological processes have been poorly characterised *ex vivo*, particularly with respect to the site and nature of antigen presentation. Antigen-presenting macrophages and B cells play a potential role in synovial inflammation, especially in mediating cartilage degradation [17,18]. However, the role played by various subsets of dendritic cells (DCs) is less clear [19]. Although enrichment of DCs in RA synovial tissue and fluid and in seronegative synovial fluid has been reported [20-26], the considerable phenotypic, morphologic and functional variations between DC subsets related to lineage, stage of maturation and tissue localisation remains less well understood. This partly reflects the difficulty in adequately identifying DC subsets in peripheral blood (PB) and tissues where most methodologies rely on complex combinations of surface markers.

Novel markers that resolve these issues, useful in human DC studies, have been recently defined [27]. Five distinct subsets of lineage-negative HLA-DR^+ ^DCs have been classified in humans. Four subsets – CD1b/c^+^, CD16^+^, blood dendritic cell antigen (BDCA)-3^+ ^and CD34^+ ^– are myeloid-lineage derived, while the fifth subset – CD123 (IL-3Rα^+^)/CD303 (BDCA-2)^+ ^– has a lymphoid phenotype and is described as a plasmacytoid DC (pDC) [27,28]. Comprising 0.5–1.0% of all circulating mononuclear cells, myeloid DCs (mDCs) are characterised as CD1c^+^/CD11c^+^/CD45RO^+^/HLA-DR^+^/CD123 (IL-3Rα)^lo^. Capable of IL-12-p70, IL-6, TNF-α and IL-10 production in response to bacterial or CD40L stimulation as well as antigen capture and presentation [29], mDCs require granulocyte–macrophage colony-stimulating factor (GM-CSF) for survival *in vitro *and can differentiate into interstitial DCs and Langerhans cells in the presence of GM-CSF, IL-4 and tumour growth factor beta [30]. Conversely pDCs, characterised as CD303 (BDCA-2)^+^/CD304 (BDCA-4)^+^/CD123 (IL-3Rα)^high^/CD11c^-^/HLA-DR^+^/CD45RA^+^, comprise less than 0.3% of all circulating mononuclear cells and secrete large amounts of the type I interferons (IFNs) in response to herpes or influenza virus stimulation [31,32].

It has been suggested that DCs play a role in the initiation and perpetuation of inflammatory arthritis by presentation of arthritogenic antigens to autoreactive T cells [19,33-35]. This presentation may drive aberrant memory T cell responses, promoting B cell activation and immunoglobulin class switching. DC infiltration into the synovium occurs early in the disease pathology [19,36,37]. DCs could represent important cellular targets in inflammatory synovitis, by virtue of their potent antigen presentation potential and their capacity to promote local inflammation via toll-like receptor (TLR) expression [38,39] and cytokine release [31,40-43]. Recent advances in identifying DC subsets have improved the resolution of subset heterogeneity. We therefore utilised robust assays to quantify and characterise the distribution and phenotype of mDCs and pDCs in blood from PsA patients and RA patients in comparison with osteoarthritis (OA) patients and healthy donors and synovial fluid (SF) from PsA patients and RA patients. We also investigated the functional capabilities of mDCs and pDCs isolated from SF to mature and release cytokines upon TLR stimulation.

## Materials and methods

### Patients and controls

Inflammatory arthritis patients fulfilled the American College of Rheumatology (formerly the American Rheumatism Association) criteria for RA [44], or met diagnostic criteria for PsA as previously described [45,46]. All patients and healthy donors gave informed consent and the study protocol was approved by the Ethical Committee, Glasgow Royal Infirmary. PB was obtained from patients with RA (*n *= 12), from patients with PsA (*n *= 13) and from patients with OA (*n *= 11), and was compared with healthy controls (*n *= 12). SF was obtained from a subset of these donors (RA, *n *= 6; PsA, *n *= 6). All cell analysis was undertaken on freshly isolated cells.

For cytokine investigation, cell-free SF and supernatants from cell culture were stored in the Centre for Rheumatic Disease Biobank at -70°C until analysis. Patients were attending the Centre for Rheumatic Diseases, Glasgow Royal Infirmary. At the time of the study, RA patients were receiving methotrexate (*n *= 7), sulphasalazine (*n *= 8) or hydroxychloroquine (*n *= 4), or a combination of these agents (*n *= 5). PsA patients were receiving either methotrexate (*n *= 11) or sulphasalazine (*n *= 4), or a combination of these agents (*n *= 2). OA patients did not receive any standard immune modulatory therapy. Patients on oral steroids were excluded from the study.

### Enumeration of DC subsets in PB and SF

Circulating DC populations were identified by flow cytometry using the FACScalibur system (Becton Dickinson, San Jose, CA, USA) and using a circulating DC enumeration kit (Miltenyi Biotech, Bergisch Gladbach, Germany) according to the manufacturer's instructions (Blood Dendritic Cell Enumeration Kit; Miltenyi Biotech). Briefly, a white blood cell (WBC) count was calculated from PB and SF samples using a Coulter Counter (Beckman Coulter, Fullarton, CA, USA). Aliquots of whole blood were labelled with a cocktail of antibodies including anti-CD14-phycoerythrin (PE).Cy5 and anti-CD19-PE.Cy5 plus or minus anti-CD1c-PE (mDC marker) and CD303 (BDCA-2)-fluorescein isothiocyanate (FITC) (pDC marker) or mouse IgG_2a_-PE and mouse IgG_1_-FITC antibodies. A proprietary dead cell discriminator was also included. After incubation, red cell lysis and washing, cells were fixed and examined using a FACScalibur and were analysed using Cellquest software (both Becton Dickinson). Results are expressed as the percentage of mDCs or pDCs in WBCs or as absolute numbers per millilitre of PB/SF (calculated as [% positive - % negative cells] × WBCs per ml [×10^6^/100]).

### Isolation of pDCs and mDCs from PB and SF

Both PB and SF samples were diluted in phosphate-buffered saline and mononuclear cells isolated by density gradient centrifugation over histopaque^®^-1077 (Sigma-Aldrich, Poole, Dorset, UK) and pDCs and mDCs sequentially purified by magnetic cell sorting (Miltenyi Biotec) using a high gradient Mini-MACS^® ^device. pDCs were purified by positive selection using anti-CD304 (BDCA-4)-conjugated magnetic microbeads (Miltenyi Biotec). B cells were depleted using anti-CD19-conjugated magnetic microbeads (Miltenyi Biotec) prior to mDC purification via incubation with biotin-conjugated anti-CD1c and anti-biotin-conjugated magnetic microbeads (both Miltenyi Biotec). FACScan analysis determined that the isolated cell populations from PB and SF were >95% and >93% pure, respectively (<10% neutrophil contamination).

Isolated DCs were phenotyped using the following monoclonal antibodies: CD1c-FITC (mDCs only), CD303 (BDCA-2)-FITC (pDCs only) and CD123-PE (pDCs only) (all Miltenyi Biotec), CCR5-PE (R&D Systems, Minneapolis, MN, USA) (pDCs only), and CD11c-PE (mDCs only), CD14-PE (mDCs only), CD40-APC, CD62L-FITC, CD80-FITC, CD83-APC, CD86-APC and CCR7-PE (all BD Biosciences Pharmingen, San Diego, CA, USA).

### mDC and pDC stimulation

All cells were cultured in RPMI-1640 supplemented with 5% human AB serum, L-glutamine (2 mM), penicillin (100 IU/ml), streptomycin (100 μg/ml) and amphotericin B (1.25 μg/ml) (all Sigma-Aldrich). Isolated mDCs were incubated in triplicate at (1–5) × 10^5^/ml containing 50 ng/ml GM-CSF (StemCell Technologies, Vancouver, BC, Canada) alone, or supplemented with *Staphylococcus aureus *peptidoglycan (PGN) at 10 μg/ml or CpG oligodeoxynucleotide (ODN) 2216 (ggGGTCAAGCTTGAgggggG) at 3.2 μg/ml (both InVivogen, San Diego, CA, USA). Isolated pDCs were incubated in triplicate at (1–5) × 10^5^/ml containing 20 ng/ml IL-3 (StemCell Technologies) alone or supplemented with CpG ODN 2216 at 3.2 μg/ml or ODN 2216c (control for CpG ODN 2216) (ggGGGAGCATGCTCgggggG) at 3.2 μg/ml (both InVivogen). cultures were centrifuged after 24 hours, supernatants collected and stored at -20°C until analysis, and phenotypical analysis by FACScalibur was performed as stated earlier.

### Cytokine analysis

Levels of IFN-α in pDC culture supernatants and SF samples were measured using a human IFN-α ELISA (Bender MedSystems™, San Bruno, CA, USA) according to the manufacturer's instructions. Levels of IL-10 and TNF-α in mDC and pDC culture supernatants were assessed using a Luminex kit (BioSource, Nivelles, Belgium) according to the manufacturer's instructions. Results of all cytokine analysis were normalised to 5 × 10^4 ^cells per sample.

### Statistical analysis

Data are expressed as the median and interquartile range. Statistical significance was determined using the nonparametric Mann-Whitney *U* test between disease groups and healthy controls. C-reactive protein (CRP) correlation to PB DC numbers was analysed by Spearman Rank correlation. *P *< 0.05 was considered significant.

## Results

### Numbers and proportion of circulating pDCs decreased in PsA BP and RA PB, and mDCs decreased in RA PB

The synovial compartment has been the focus of many previous studies of RA and PsA [16,47]. We chose to examine both the systemic and local levels of circulating DC subsets in inflammatory arthritis, as changes in DC subpopulations have been identified in other autoimmune diseases, particularly systemic lupus erythematosus [41,48-50]. PB was obtained from RA patients (*n *= 12) and PsA patients (*n *= 13) and was compared with PB from OA patients (*n *= 11) and healthy controls (*n *= 12).

**Figure 1:**
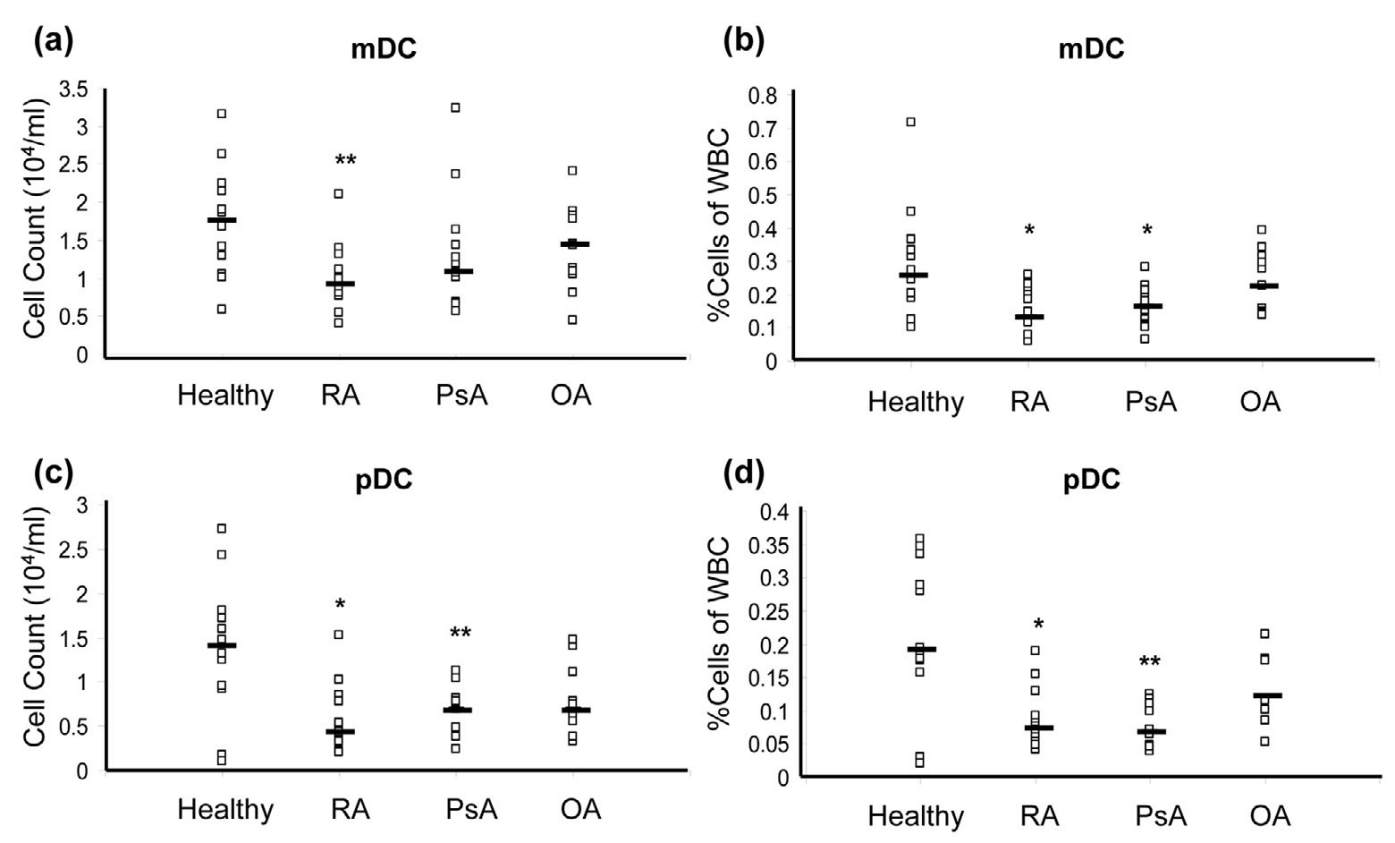
Enumeration of dendritic cell subsets in various arthritides and healthy volunteers. Plasmacytoid dendritic cells (pDCs) and myeloid dendritic cells (mDCs) are significantly decreased in rheumatoid arthritis (RA) peripheral blood (PB), and pDCs are significantly decreased in psoriatic arthritis (PsA) PB. The total number of (**a**) mDCs and (**c**) pDCs per millilitre of PB and the percentage of (**b**) mDCs and (**d**) pDCs of white blood cells (WBC) in healthy subjects (*n *= 12), RA subjects (*n *= 12), PsA subjects (*n *= 13) and osteoarthritis (OA) subjects (*n *= 11). Squares indicate individual samples. -, median. *P < 0.05, **P < 0.01.

In RA PB, mDCs were significantly decreased compared with healthy controls, expressed as total numbers per millilitre of PB (Figure 1a) (*P *= 0.0086) and as a percentage of WBCs (Figure 1b) (*P *= 0.0262). In PsA PB, mDCs were not significantly decreased compared with healthy controls when expressed as total numbers per millilitre of PB (*P *= 0.1147) but were significantly decreased when adjusted to the percentage of WBCs (*P *= 0.018), most probably reflecting an elevation in another cell population as indicated by a significantly increased WBC count in PsA PB compared with healthy controls (Table 1, *P *= 0.0470).

In contrast, circulating pDCs were significantly decreased in both RA PB and PsA PB compared with healthy controls expressed either as total numbers per millilitre of PB (Figure 1c) (RA, *P *= 0.0194; PsA, *P *= 0.0098) or indeed as a percentage of WBCs (Figure 1d) (RA, *P *= 0.0180; PsA, *P *= 0.0051). In OA PB, although a trend towards a decrease in pDC numbers per millitre of PB was observed, neither mDC nor pDC numbers were significantly reduced compared with healthy controls whether data were expressed as a percentage of total WBCs or as total cell numbers per millilitre of PB.

### Reduced RA PB mDC numbers are inversely correlated to CRP

**Figure 2:**
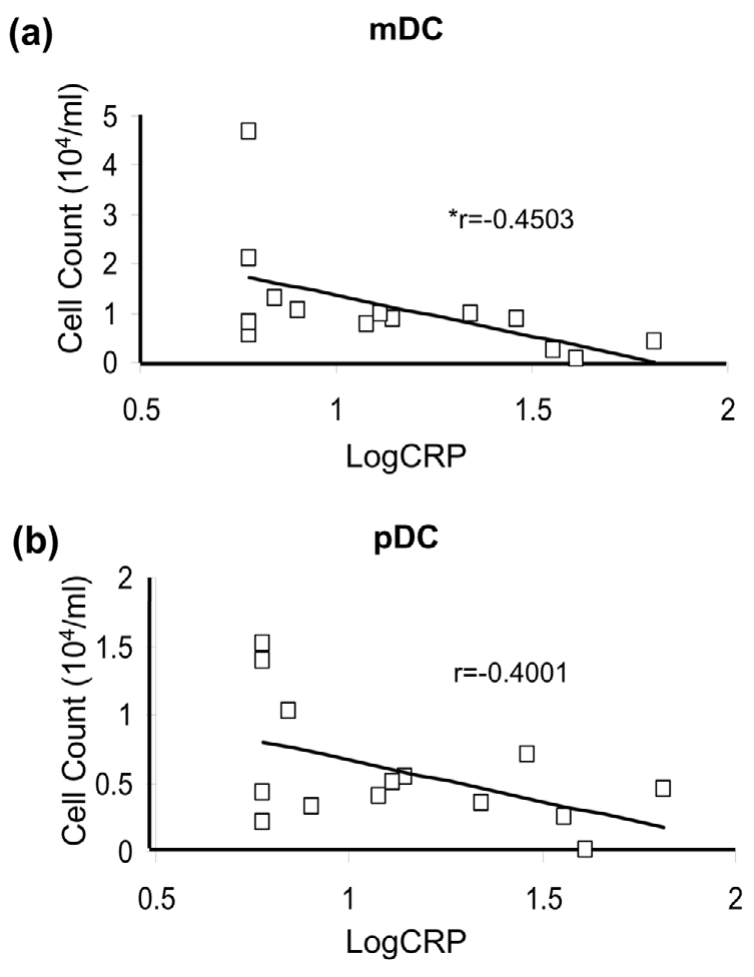
Relationship of DC numbers to acute phase response. Circulating myeloid dendritic cell (mDC) numbers are significantly inversely correlated to C-reactive protein (CRP) levels in rheumatoid arthritis (RA) peripheral blood (PB). The CRP levels of RA patients (*n *= 12) compared with total numbers of (**a**) mDCs and (**b**) plasmacytoid dendritic cells (pDCs) per millilitre of PB. CRP levels are calculated on a logarithmic scale, and squares indicate individual samples. *P < 0.05.

The significant decrease of pDCs and mDCs in RA PB and of pDCs in PsA PB, as well as the trend towards a decrease in pDCs in OA PB, were intriguing and led us to investigate a possible correlation to the magnitude of inflammatory disease activity. Accordingly, we analysed pDC and mDC numbers from PB of RA patients, PsA patients and OA patients compared with serum CRP concentrations. The mDC numbers per millilitre of PB, but not the pDC numbers per millilitre, were significantly inversely correlated to CRP levels from RA patients (*P *= 0.021, *r *= -0.45233 and *P *= 0.228, *r *= -0.4001, respectively) (Figure 2a,b). Neither mDC nor pDC numbers per millilitre of PB were significantly inversely correlated to serum CRP concentrations from PsA patients or OA patients. This could reflect the relatively poorer utility of CRP as a surrogate for disease activity in the latter conditions.

### CD62L is aberrantly expressed on RA and PsA circulating PB DCs

**Figure 3:**
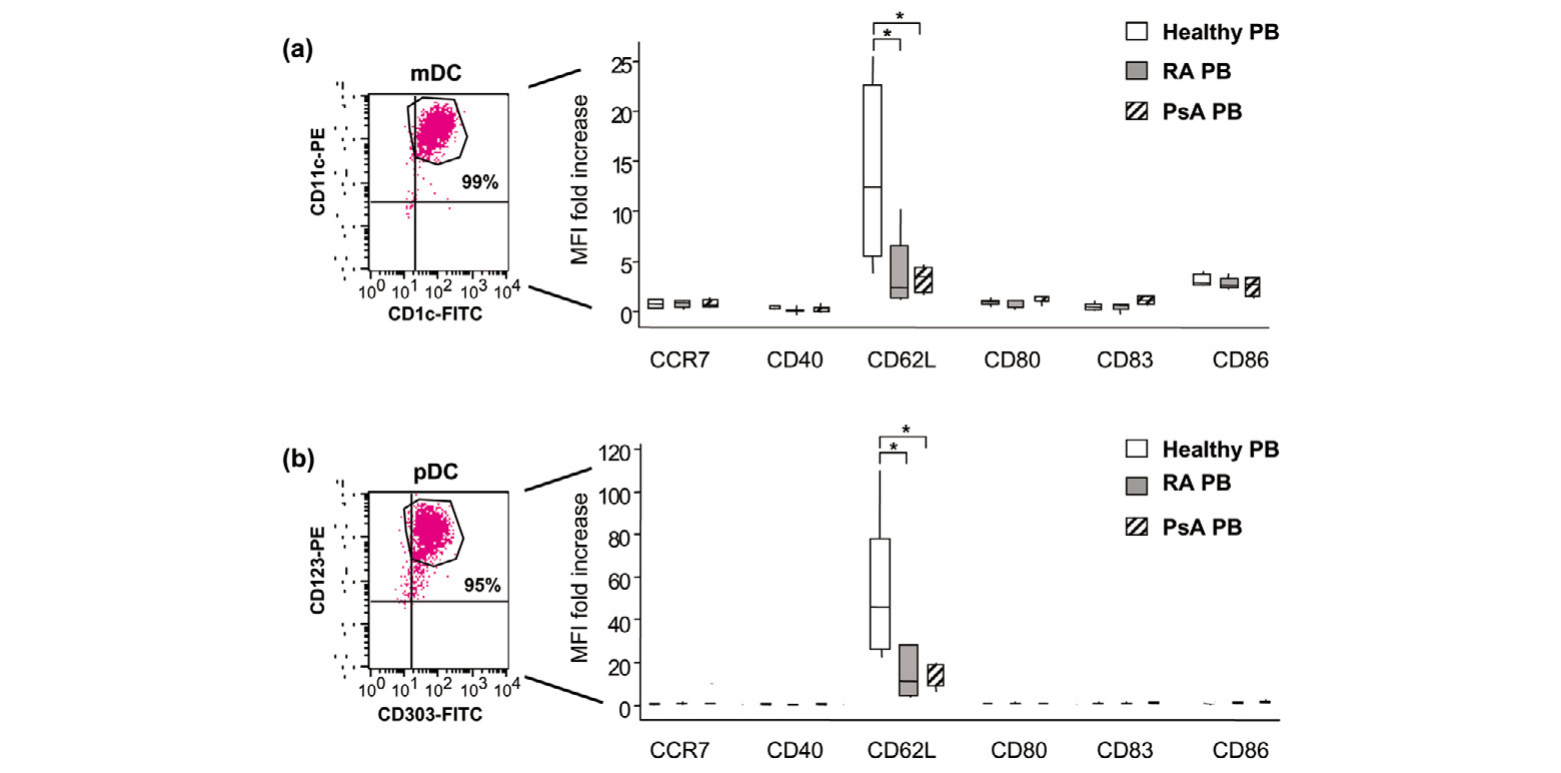
Phenotypic characterisation of blood derived DC subsets. Rheumatoid arthritis (RA) and psoriatic arthritis (PsA) peripheral blood (PB) plasmacytoid dendritic cells (pDCs) and myeloid dendritic cells (mDCs) have an immature phenotype and significantly decreased CD62L expression. (**a**) Migration and maturation markers on CD1c^+^/CD11c^+ ^mDCs purified from RA PB (*n *= 5) and from PsA PB (*n *= 5) compared with healthy control PB (*n *= 5). (**b**) CD303 (BDCA-2)^+^/CD123^+ ^pDCs purified from RA PB (*n *= 5) and from PsA PB (*n *= 5) compared with healthy control PB (*n *= 5). Values are calculated as the mean fluorescent intensity (MFI) fold of the increase above the isotype control. The median and interquartile range is shown. *P < 0.05.

To further study the phenotype of PB mDCs and pDCs, these cells were isolated in a subset of RA PB (*n *= 5) and PsA PB (*n *= 5) and were compared with healthy controls (*n *= 5). The purity of the isolated DCs was confirmed by double staining with CD1c and CD11c for mDCs (Figure 3a) and with CD303 (BDCA-2) and CD123 for pDCs (Figure 3b); this purity was routinely >99% mDCs and >95% pDCs. Fluorescence-activated cell sorting analysis indicated that both CD1c^+^/CD11c^+ ^mDCs and CD303 (BDCA-2)^+^/CD123^+ ^pDCs from PsA PB and RA PB displayed an immature phenotype similar to mDCs and pDCs isolated from healthy controls (Figure 3a,b, respectively), with absent expression of the maturation marker CD83 and low to absent expression of CD40, CD80 and CD86 and the chemokine receptor CCR7. However, expression of the adhesion molecule CD62L (L-selectin) was significantly reduced on inflammatory arthritis-derived mDCs (RA, *P *= 0.0371; PsA, *P *= 0.0122) and pDCs (RA, *P *= 0.0367; PsA, *P *= 0.0373) compared with healthy control PB mDCs and PB pDCs, respectively.

### RA SF and PsA SF contain mDCs and pDCs

The aforementioned data strongly suggest that DC subset distribution in inflammatory arthritis is abnormal and commensurate with either enhanced migration to the synovial compartment or reduced release from bone marrow. To investigate the former possibility, we chose to evaluate DC subsets in SF. To this aim, we enumerated mDCs and pDCs in SF and matched PB obtained from six patients with RA and from six patients with PsA.

**Figure 4:**
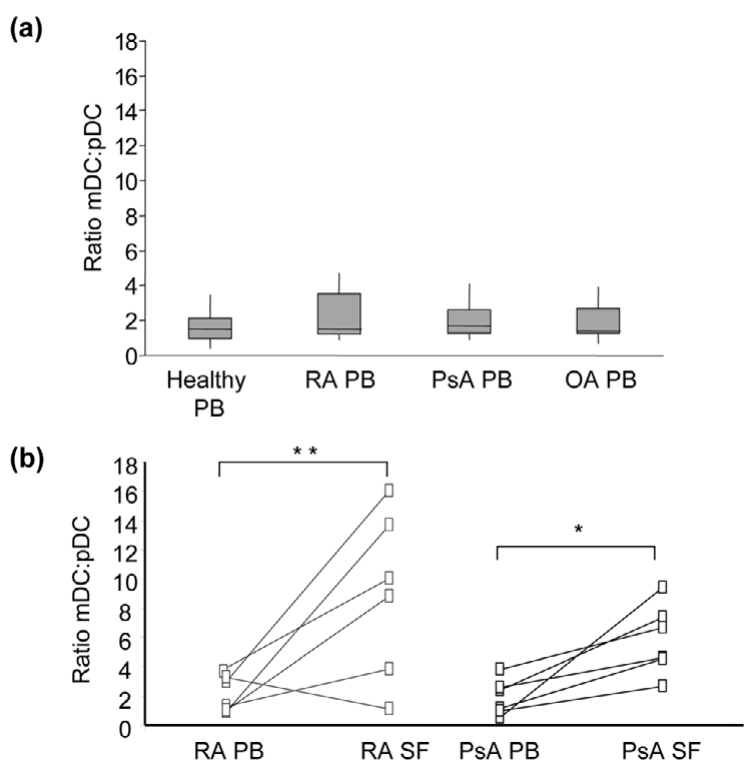
Ratios of DC subsets in tissue compartments. Myeloid dendritic cell/plasmacytoid dendritic cell (mDC:pDC) ratios in synovial fluid (SF) are significantly increased when compared with matched peripheral blood (PB). (**a**) The mDC:pDC ratio in PB from rheumatoid arthritis (RA) subjects (*n *= 12), psoriatic arthritis (PsA) subjects (*n *= 13) and osteoarthritis (OA) subjects (*n *= 11) was not significantly altered compared with healthy control subjects (*n *= 12); median and interquartile range are shown. (**b**) The mDC:pDC ratio in SF from RA subjects (*n *= 6) and PsA subjects (*n *= 6) was significantly increased compared with matched PB samples. Squares indicate individual matched samples. The ratio was calculated from total number of cells per millilitre. *P < 0.05, **P < 0.01.

Magnetic sorting and flow cytometry revealed that, whereas absolute mDC and pDC numbers varied greatly between individual patients, both were consistently present in RA SF and PsA SF (Table 2). The mDC:pDC ratio in PB was approximately 2:1 in both RA patients and PsA patients, and was similar to that seen in OA patients and healthy controls (Figure 4a). In contrast, this ratio was significantly increased in both RA SF (median, 9.4:1; *P *= 0.0082) and PsA SF (median, 6.05:1; *P *= 0.0453) (Figure 4b) compared with matched PB.

### SF mDCs have a semi-mature phenotype but pDCs are immature

**Figure 5:**
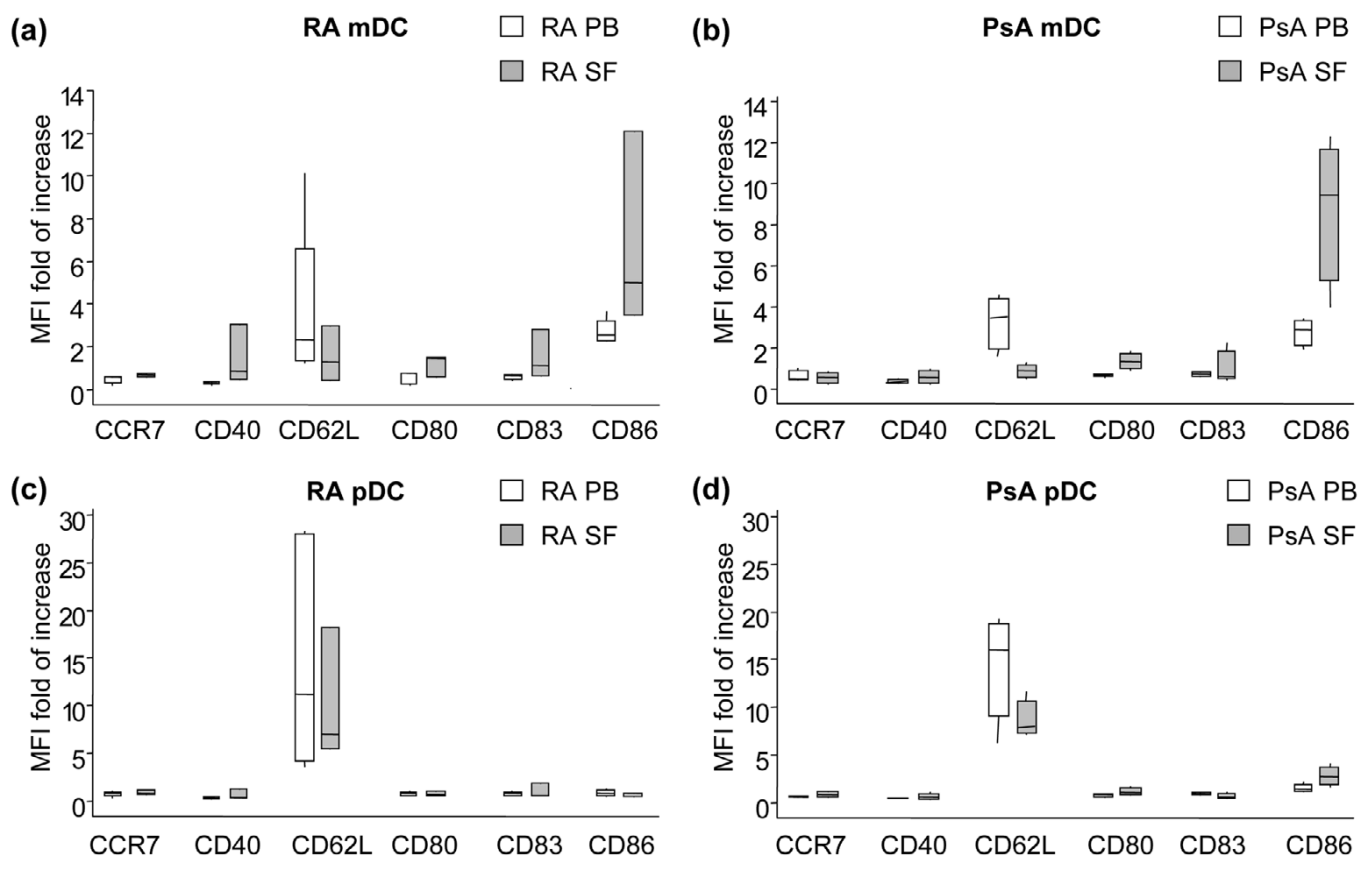
Phenotypic characterisation of synovial DC subsets. Rheumatoid arthritis (RA) and psoriatic arthritis (PsA) synovial fluid (SF) plasmacytoid dendritic cells (pDCs) have an immature phenotype, but myeloid dendritic cells (mDCs) have a semi-mature phenotype. Migration and maturation markers on CD1c^+^/CD11c^+ ^mDCs purified from (**a**) RA SF (*n *= 3) compared with RA peripheral blood (PB) (*n *= 5) and (**b**) PsA SF (*n *= 3) compared with PsA PB (*n *= 5), and CD303^+^/CD123^+ ^pDCs purified from (**c**) RA SF (*n *= 3) compared with RA PB (*n *= 5) and (**d**) PsA SF (*n *= 3) compared with PsA PB (*n *= 5). Values are calculated as the mean fluorescent intensity (MFI) fold of the increase above the isotype control. The median and interquartile range are shown. *P < 0.05

To investigate the phenotype of mDCs and pDCs in SF, cells were isolated from a subset of RA SF (*n *= 3) and PsA SF (*n *= 4), as stated earlier, and were analysed using a range of cell surface markers. Isolated DC subsets were routinely >90% pure, and the phenotype was determined by gating specifically on CD1c^+^/CD11c^+ ^cells (mDCs) and CD303 (BDCA-2)^+^/CD123^+ ^cells (pDCs). In both RA SF (Figure 5a) and PsA SF (Figure 5b) the phenotype of mDCs was similar, with increased expression of CD40, CD80, CD83 and CD86 in comparison with their RA PB and PsA PB counterparts, respectively, indicating a semi-mature phenotype. In contrast, RA SF (Figure 5c) and PsA SF (Figure 5d) pDCs displayed an immature phenotype, generally comparable with that of circulating PB pDCs, with low to absent expression of CD40, CD80 and CD83.

The only difference observed was an increase of CD86 expression on PsA SF pDCs compared with PsA PB pDCs, although this was not observed on RA SF pDCs. Since CD62L was significantly reduced, but not absent, on mDCs and pDCs from RA PB and PsA PB, we also evaluated expression on SF-derived cell subsets. CD62L was decreased on mDCs and pDCs from both RA SF and PsA SF DC subsets compared with RA PB and PsA PB.

**Figure 6:**
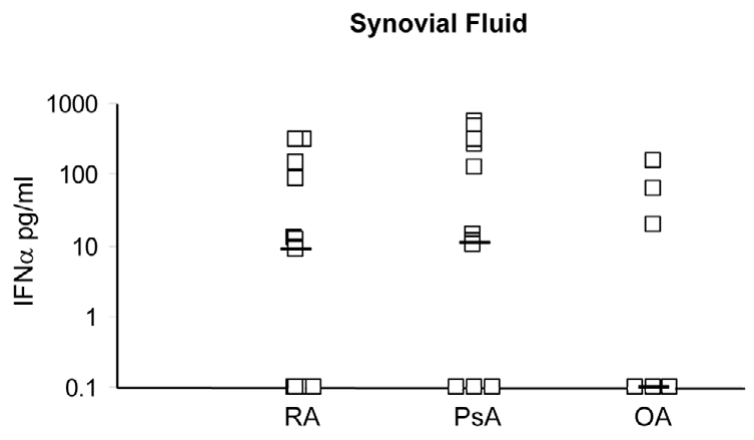
Detecting interferon alpha in synovial fluid. Rheumatoid arthritis (RA) synovial fluid (SF), psoriatic arthritis (PsA) SF and osteoarthritis (OA) SF contain interferon alpha (IFN-α). The level of IFN-α in SF from RA patients (*n *= 18), PsA patients (*n *= 15) and OA patients (*n *= 8) was measured by ELISA. Squares indicate individual samples expressed on a log scale. -, median.

The presence of relatively large numbers of immature pDCs in SF was intriguing, since immature pDCs characteristically produce high levels of IFN-α [51]. The level of IFN-α expression in inflammatory synovial fluids is little characterised and declared absent in previous publications [52]. We therefore analysed RA SF (*n *= 18), PsA SF (*n *= 14) and OA SF (*n *= 7) for the presence of IFN-α by ELISA (Figure 6). By this methodology, IFN-α was detectable in many SFs, derived not only from RA and PsA samples but also from a small number of OA SF. However, there were no significant differences between diseases.

### SF mDCs and pDCs mature and release cytokines upon TLR stimulation

It is now recognised that DCs are activated via TLRs [39]. TLR-induced cytokine production was therefore evaluated in disease DCs compared with healthy PB-derived DCs. We purified mDCs and pDCs as already mentioned and evaluated the addition of the TLR2 agonist *S. aureus *PGN (for mDCs) or the TLR9 agonist CpG ODN 2216 (for pDCs) over 24 hours. For control purposes, mDCs were also incubated with GM-CSF alone or the irrelevant TLR agonist CpG ODN 2216 (mDCs do not express TLR9), and pDCs were also incubated with IL-3 alone or with control ODN 2216c. The phenotypic changes in DC subsets were measured by fluorescence-activated cell sorting analysis and the cytokine release was measured by multiplex assay or ELISA. Irrelevant TLR agonists did not induce cytokine production in any culture conditions tested, nor did they have any effect on *in vitro *maturation of DC subsets (data not shown).

**Figure 7:**
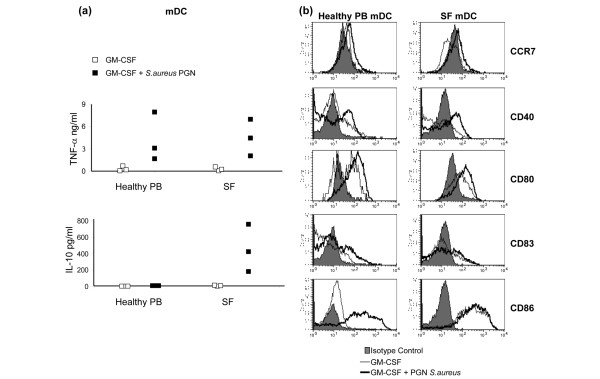
Cytokine release and phenotypic changes following mDC exposure to TLR agonist. Synovial fluid (SF) myeloid dendritic cells (mDCs) mature and release tumour necrosis factor alpha (TNF-α) at comparable levels with healthy peripheral blood (PB) mDCs, but only SF mDCs release IL-10. Cytokine release and the maturation status of mDCs purified from healthy control PB (*n *= 3) or inflammatory arthritis SF (rheumatoid arthritis, *n *= 2; psoriatic arthritis, *n *= 1) were assessed after 24-hour incubation with granulocyte–macrophage colony-stimulating factor (GM-CSF)-supplemented media alone or containing *Staphylococcus aureus *peptidoglycan (PGN) (10 μg/ml). (**a**) The concentrations of TNF-α and IL-10 in supernatant from the cultures were determined by multiplex analysis and were expressed as release per 5 × 10^4 ^cells. Squares indicate individual donors. (**b**) Maturation and co-stimulation marker expression was determined by flow cytometry, and one representative example from each of the healthy control PB and inflammatory arthritis SF groups are shown.

Distinct patterns of cytokine production emerged on comparison of PB-derived versus SF-derived mDCs and pDCs. In PGN-stimulated cultures, mDCs from both PB and SF produced comparable levels of TNF-α (Figure 7a). In contrast, SF mDCs produced considerably higher levels of IL-10 following PGN stimulation than did the PB comparators. IL-12 p70, IL-2 and IL-4 levels were below the limit of sensitivity of the assay in all cultures. Furthermore, coincident addition of PGN with GM-CSF induced higher levels of maturation marker expression (compared with GM-CSF alone), particularly CCR7, CD40, CD80 and CD83 (Figure 7b).

**Figure 8:**
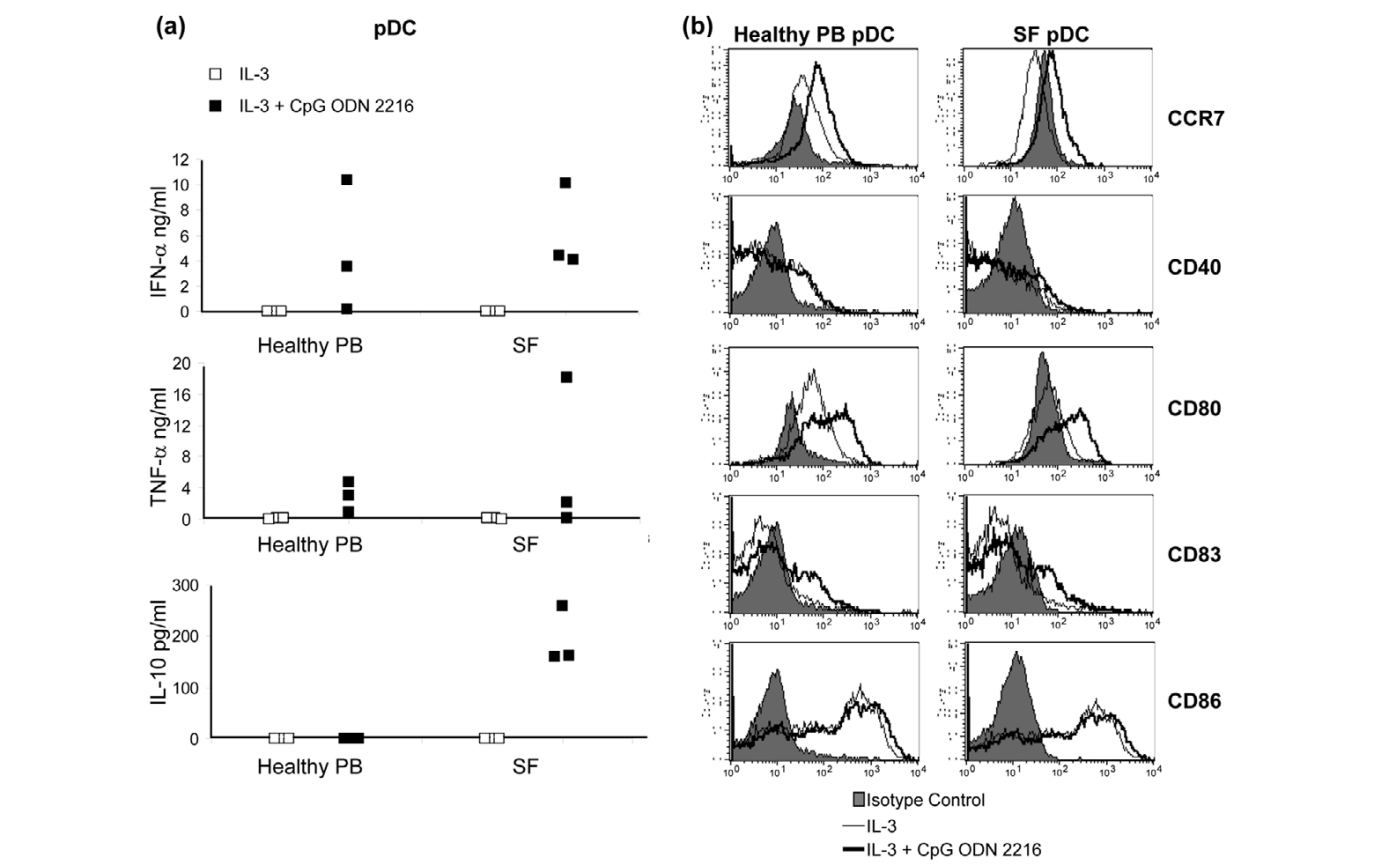
Cytokine release and phenotypic changes following pDC exposure to TLR agonst. Synovial fluid (SF) plasmacytoid dendritic cells (pDCs) mature and release interferon alpha (IFN-α) and tumour necrosis factor alpha (TNF-α) at comparable levels with healthy control peripheral blood (PB) pDCs, but only SF pDCs release IL-10. Cytokine release and the maturation status of pDCs purified from healthy control PB (*n *= 3) and inflammatory arthritis SF (rheumatoid arthritis, *n *= 2; psoriatic arthritis, *n *= 1) were assessed after 24-hour incubation with IL-3-supplemented media alone or containing the TLR9 agonist CpG ODN 2216 (3.2 μg/ml). (**a**) The concentrations of IFN-α, TNF-α and IL-10 in supernatant from cultures was determined by multiplex and ELISA analysis, and was expressed as release per 5 × 10^4 ^cells. Squares indicate individual donors. (**b**) Maturation and co-stimulation marker expression was determined by flow cytometry, and one representative example from each of the healthy control PB and inflammatory arthritis SF groups are shown.

We next examined the cytokine release by pDCs. CpG ODN 2216 stimulated the release of large amounts of both IFN-α and TNF-α, from both PB-derived and SF-derived DCs. However, only SF pDCs produced IL-10 following CpG ODN 2216 stimulation (Figure 8a). Neither IL-2 nor IL-4 release was detected under these conditions. Upon phenotypic analysis, the upregulation of CCR7, CD80 and CD83 (Figure 8b) after CpG ODN 2216 stimulation was observed. Finally, we noted that growth factor alone, namely GM-CSF or IL-3, was sufficient to promote maturation marker expression but did not induce cytokine production.

Together these data indicate that SF DC subsets retain the capacity to upregulate maturation markers and respond to TLR agonists, and that the latter are necessary to promote optimal maturation and cytokine production.

## Discussion

The relative role of innate and adaptive components of the host immune response to inflammatory synovitis is of considerable current interest. DCs comprise a heterogeneous network of professional antigen-presenting cells, capable not only of presenting antigen and inducing subsequent adaptive immune responses, but also of sensing the inflammatory environment and contributing to the cytokine milieu therein. While implicated in the pathogenesis of RA and PsA [19,33-37], prior analysis of DC subsets has been hampered by a lack of specific DC markers and reliable quantitation methodologies. The present study has utilised the pDC-specific marker CD303 (BDCA-2) and the mDC marker CD1c within a sensitive assay system to provide enumeration and phenotypic analysis of the DC subsets in PB and SF from PsA patients and RA patients. Thereafter we have utilised novel markers to purify DC subpopulations and perform a functional evaluation of their activation potential *ex vivo*. Finally we have for the first time attempted a phenotypic description of DC subsets in PsA and compared them with those in RA.

We observed a reduction in circulating PB pDC and PB mDC populations in RA patients, and in pDC populations in PsA patients. That this was not observed in prior investigation probably reflects less sensitive methodologies [52,53] and the identification of subsets based upon CD123 expression, which is also expressed by basophils [54], haematopoietic progenitor cells [55], endothelial cells [56], monocytes, eosinophils and small subsets of lymphocytes [57]. The observed decrease in DC numbers is not due to steroid therapy, which has been reported to reduce DC numbers [58] and function [59], as patients on corticosteroids were excluded from the study.

Decreased PB DC numbers most probably reflect DC migration from the circulation and accumulation to the inflamed synovial compartment. This is consistent with previous studies [52,53,60] and with our own data, indicating comparatively high numbers of mDCs and pDCs in SF effusions from both PsA patients and RA patients. In addition, the SF DC subset expression of CD62L (normally cleaved from the cell surface after leukocyte tethering and rolling across high endothelial venules [61]) was lower in both PsA SF and RA SF compared with the peripheral blood compartment, further indicative of migration.

In preliminary studies, we have utilised these novel markers to identify pDCs in RA, PsA and in OA synovial membrane (SM) itself (see Additional file 1). Future detailed phenotypic analysis of such membrane DC subsets is now necessary, and will offer insight into the migratory pathway and functional activities of distinct DC subsets in chronic synovitis. Finally, the trend towards decreased PB pDC numbers in the OA patients, although not significant, may be indicative of altered DC migration toward the synovial compartment. OA is associated with synovial hypertrophy, hyperplasia, local inflammation and recurrent effusions [62].

Other factors may operate in regulating detectable blood DC numbers, however, particularly within the bone marrow – in which it is increasingly recognised that functional maturation defects may exist for leukocyte lineages [63]. For example, reduced pDC numbers in circulating PB could reflect elevated TNF-α expression [64,65]: TNF-α is known to inhibit fms-like tyrosine kinase 3-ligand-driven differentiation of pDC from bone marrow progenitors [51,66]. Future studies will be required to establish the circulating half-life and tissue destination of DC subsets in the context of inflammatory arthropathies, and indeed of the effect of cytokine blockade on such parameters. It should also be noted that reduced circulating DC subsets have been demonstrated in several other inflammatory disorders, including systemic lupus erythematosus [48] and chronic active hepatitis [67], and that regulatory pathways in such diseases may also be informative in elucidating DC function in synovitis.

A further novel observation was the reduced CD62L expression on both DC subsets in PsA PB and RA PB. Reduced CD62L expression on RA PB CD3^+ ^and CD19^+ ^lymphocytes, monocytes and granulocytes [68] has been linked to immunosuppressive therapies including methotrexate [69], and a similar mechanism could explain our observations. However, downregulation of adhesion molecules such as CD62L is necessary for the release of CD34^+ ^progenitors to the PB [70], and it is possible that a CD62L^lo ^PB DC population may indicate altered recirculation or modified release of bone marrow DC precursors.

Purified SF pDCs were recently reported to exhibit a relatively immature phenotype given their inflammatory environment [52,53]. Similar observations have been made for mDCs [26,60]. We now confirm these observations in RA and extend the data to include PsA-derived cells, indicating that the inflamed synovial compartment *per se *can sustain immature DCs capable by inference of antigen sensing, capture and, ultimately, presentation. Upon removal from the SF, both mDCs and pDCs were capable of increased maturation marker expression and, provided with TLR agonist activation, were capable of cytokine release. There does not therefore appear to be an intrinsic functional defect of DCs. Whereas TLR2 or TLR9 stimulation was not sufficient to induce substantial IL-10 release from healthy PB mDCs and pDCs, it was sufficient for IL-10 release from PsA SF and RA SF purified cells.

The functional implications of this observation are unclear and require further examination to include other TLR agonists and DC activation conditions. In particular we now wish to examine the cross-regulation of mDC and pDC subsets in the context of inflammatory synovitis. That said, the technical demands of isolating primary DCs from an inflamed compartment (particularly pDCs) are not insignificant and place limitations on feasible functional analyses. Very few groups have thus far attempted formal functional evaluation of purified DC subsets from any inflamed tissue. Our approach combines a broad but pragmatic approach to start to examine DC function *ex vivo*. Future technical refinements will be required to perform the desired extensive *in vitro *analyses of DC function that will more comprehensively address their functional role.

These are the first data to investigate purified DC subsets in PsA patients. Prior studies have identified pDC in cutaneous psoriasis and increased type I IFN expression in psoriatic plaques after application of a TLR agonist [71]. Numerous data indicate a role of T cells in PsA pathogenesis [72]; determining the functional role of both pDCs and mDCs in PsA synovium should therefore be informative. The present study provides the essential pre-requisite for such studies in determining the presence, enumeration and function of each subset in PB and SF.

IFN-α was detected in SF in both RA patients and PsA patients. The role for this cytokine in synovial pathogenesis is indicated by numerous reports linking IFN-α therapy to induction of RA and PsA in patients with no pre-existing clinical arthritis [73-75]. Moreover, IFN-α is sufficient to drive monocyte differentiation to mDCs in systemic lupus erythematosus patients [76]. The significantly increased mDC:pDC ratio identified in SF may indicate *in situ *differentiation of monocytes shed from the synovial lining layer to mDCs, driven in part by IFN-α in the fluid. It is therefore intriguing to consider a local cytokine-mediated feedback loop whereby pDC IFN-α release promotes local mDC maturation. Consistent with this, we have recently observed that PsA SF is capable of inducing differentiation of mDCs from PB-derived monocytes (unpublished observations).

While only mature DCs are able to induce the activation of T cells and the differentiation of B cells into antibody-producing plasma cells, immature DCs are able to capture antigen at picomolar and nanomolar concentrations [77]. In the steady state, presentation of these self-antigens should sustain tolerance. However, the presentation of these antigens within an inflammatory context, such as the inflamed synovial compartment in susceptible individuals, may lead to chronic inflammation and autoimmune disease. SF apparently contains a pool of immature DC subsets of both mDC and pDC lineage that could facilitate (auto)antigen capture, and thereby the initiation or perpetuation (neoepitopes) of autoimmunity.

## Conclusion

In summary, we have performed a comparative evaluation of RA and PsA circulating and SF DC subsets. Our data show that mDC and pDC numbers are significantly decreased in RA PB and that pDCs are significantly decreased in PsA PB, and that both subsets exhibit reduced CD62L expression. These data indicate an altered recirculation or circulating half-life for DC subsets in inflammatory arthritis. Both mDCs and pDCs are present in SF from RA and PsA, with pDCs expressing an immature phenotype whereas mDCs express a semi-mature phenotype. Synovial fluid DCs remain responsive to TLR agonism, exhibiting maturation *ex vivo *and cytokine release. Future studies are now required to evaluate those factors that prevent local maturation of DCs in synovial fluid and to determine the functional significance of immature DCs maintained in the fluid compartment of the inflamed joint.

## Abbreviations

BDCA = blood dendritic cell antigen; CCR = cc-chemokine receptor; CpG = immunostimulatory bacterial CpG-DNA sequence; CRP = C-reactive protein; DC = dendritic cell; ELISA = enzyme-linked immunosorbent assay; FITC = fluorescein isothiocyanate; GM-CSF = granulocyte–macrophage colony-stimulating factor; IFN = interferon; IL = interleukin; mDC = myeloid dendritic cell; OA = osteoarthritis; ODN = oligodeoxynucleotide; PB = peripheral blood; pDC = plasmacytoid dendritic cell; PE = phycoerythrin; PGN = peptidoglycan; PsA = psoriatic arthritis; RA = rheumatoid arthritis; SF = synovial fluid; TLR = toll-like receptor; TNF = tumour necrosis factor; WBC = white blood cell.

## Competing interests

The authors declare that they have no competing interests.

## Authors' contributions

SLJ, MCL, PPT and IBM conceived the study design. SLJ carried out the DC enumeration, purification, cell culture, flow cytometry, ELISA and statistical analysis. ARF participated in the flow cytometry, and ARF and JAG carried out the Luminex analysis. RDS and IBM supplied clinical samples. SLJ, MCL and IBM wrote the manuscript.

## Supplementary Material

Additional file 1 Immunohistochemical analysis of synovial tissue samples. Click here for file

## Figures and Tables

**Table 1:** Patient data

	Characteristic	Peripheral blood study group	Synovial fluid study group
Rheumatoid arthritis	Age [mean ± SD (range)]	58 ± 12 (24–80)	59 ± 15 (33–70)
	Female:male	10:2	5:1
	White blood cells [mean ± SEM]	6.4 × 10^6 ^± 1.2 × 10^4^/ml	
	C-reactive protein	26 ± 6 (6–100)	22 ± 7 (6–41)
Psoriatic arthritis	Age [mean ± SD (range)]	42 ± 14 (24–81)	41 ± 15 (25–50)
	Female:male	10:3	5:1
	White blood cells [mean ± SEM]	8.0 × 10^6^* ± 4.5 × 10^5^/ml	
	C-reactive protein	14 ± 4 (6–73)	30 ± 15 (6–73)
Osteoarthritis	Age [mean ± SD (range)]	63 ± 12 (52–82)	
	Female:male	4:6	
	White blood cells [mean ± SEM]	6.0 × 10^6 ^± 8.9 × 10^5^/ml	
	C-reactive protein	10 ± 3 (6–14)	
Healthy	Age [mean ± SD (range)]	44 ± 11	
	Female:male	6:6	
	White blood cells [mean ± SEM]	6.4 × 10^6 ^± 4.7 × 10^5^/ml	

SD, standard deviation; SEM, standard error of the mean.

*P < 0.05 compared with healthy control peripheral blood.

**Table 2:** Absolute number of cells (×10^4^/ml) in rheumatoid arthritis and psoriatic arthritis peripheral blood and matched synovial fluid

	White blood cells	Myeloid dendritic cells	Plasmacytoid dendritic cells
Rheumatoid arthritis (*n *= 6)			
Peripheral blood	652 ± 93.7	1.36 ± 0.641	0.582 ± 0.211
Synovial fluid	1020 ± 235	11.6 ± 3.12	3.24 ± 1.71
Psoriatic arthritis (*n *= 6)			
Peripheral blood	969 ± 240	1.39 ± 0.420	0.761 ± 0.220
Synovial fluid	2860 ± 351	37.5 ± 8.72	7.20 ± 2.23

Data presented as the mean ± standard error of the mean.
