# Supplementary material for: Enumeration and phenotypical analysis of distinct dendritic cell subsets in psoriatic arthritis and rheumatoid arthritis
Source: Arthritis Res Ther. 2005 Dec 16;8(1):R15. doi: 10.1186/ar1864 (PMC1526567; doi:10.1186/ar1864)

Additional Data File 1

Figure 1

CD1c$^+$ mDC and CD304$^+$/CD123$^+$ pDC are present in RA, PsA and OA SM. Single stain CD1c$^+$ mDC (stained red) and double stained CD304$^+$/CD123$^+$ pDC (stained black) were identified in the inflammatory cell infiltrate to the sub-lining layer and around vessels of synovial membrane of RA, PsA and OA SM as assessed by immunohistochemical analysis. Examples of positive cells are indicated by red arrows. One example of each disease type is shown with isotype control.

Method

Synovial tissue samples

For synovial tissue collection small-bore arthroscopy was performed under local anesthesia and synovial tissue samples were obtained from multiple sites in the joint using 2-mm grasping forceps. From some patients, synovial tissue biopsies were obtained after synovectomy or arthroplasty. Synovial biopsy samples were collected and snap-frozen in TissueTek OCT (Miles, Elkhart, IN). Frozen blocks were stored at -70°C until sectioned for staining. Sections (5 μm) were cut in a cryostat and mounted on Superfrost® Plus microscope slides (BDH Laboratory Supplies, Poole, UK) that were stored at -70°C until use for immunohistochemical analysis. See Table 1 for patient and control details.

Immunohistochemical staining

Acetone-fixed cryosections were incubated in 0.5%H_2O_2/methanol for 30 min followed by blocking in 20% horse serum/ 20% human serum/PBS for 30 minutes, all at room temperature. Sections were then incubated with mAbs against CD1c (for detection of mDC) (Beckman Coulter, Fullerton, CA) or CD123 (IL-3Rα) (for detection of pDC) (BD Biosciences Pharmingen) for 90 min at room temperature. As negative controls isotype-matched antibodies were applied (Mouse IgG_1, Dakocytomation, Glostrup, Denmark). After washing, slides were incubated with biotinylated horse anti-mouse IgG (H+L) (Dakocytomation). Slides were then incubated with Vectastain ABC (Vector, Burlingame, CA) for 30 min followed by 5 min incubation with NovaRED™ (Vector) for CD1c sections or DAB Ni (Vector) for CD123 sections. With this procedure $CD1c^+$ mDC stained red and $CD123^+$ cells stained grey. For CD1c stained slides, other cellular elements were counterstained with Harris modified haematoxylin solution (Sigma-Aldrich), followed by alcohol dehydration and mounting in DPX mountant for microscopy (BDH Laboratory Supplies). CD123 stained sections were instead incubated for 90 min at room temperature with CD304 (BDCA-4) (Miltenyi Biotec). Immunohistochemistry was then carried out as described above, but using NovaRED™ (Vector). By this procedure CD304 (BDCA-4)$^+$ cells were stained red, with CD304 (BDCA4)$^+$/$CD123^+$ cells identified as black.

Additional Data Figure 1

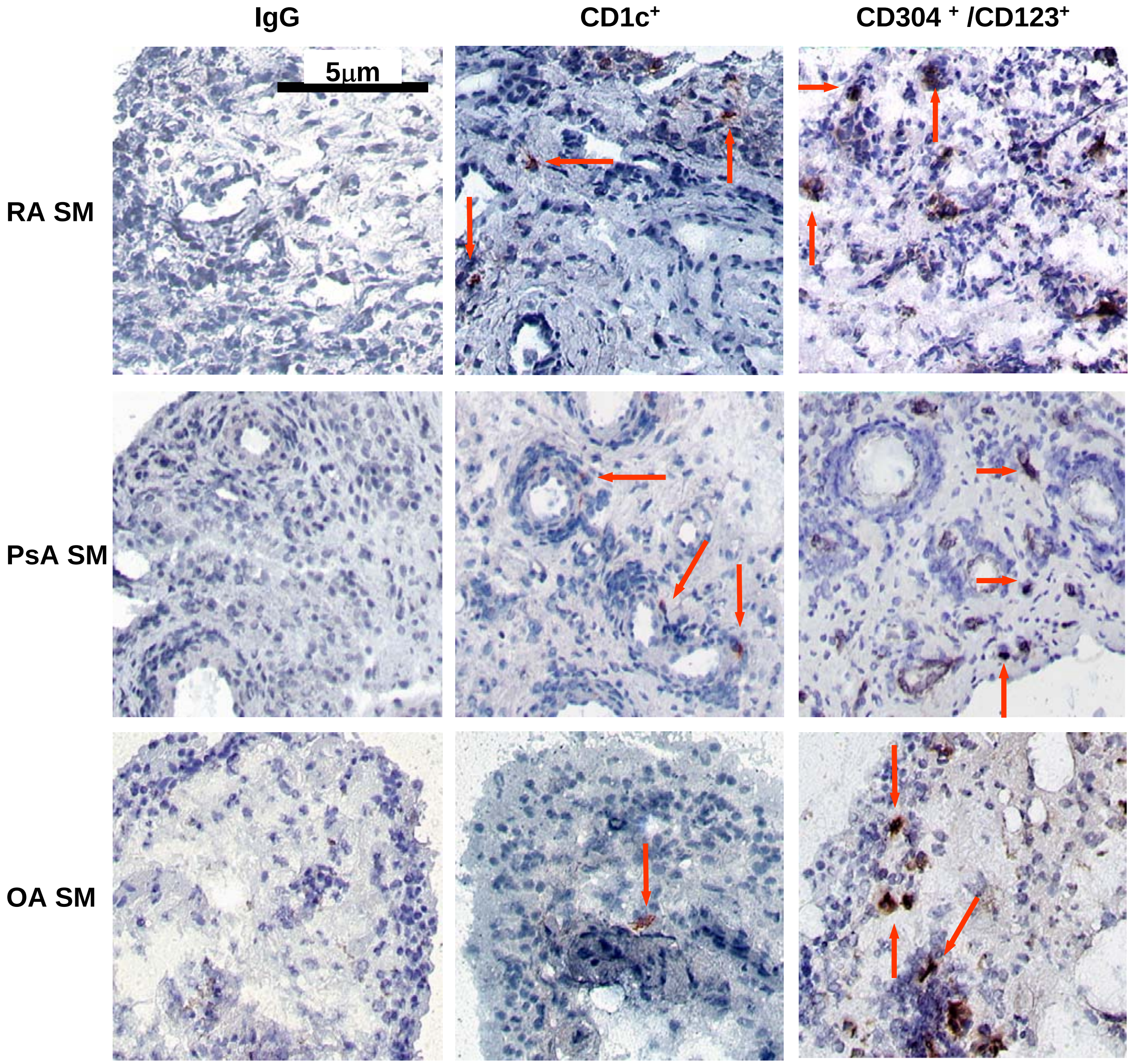

Supplement: Additional file 1 — Immunohistochemical analysis of synovial tissue samples. [file ar1864-S1.pdf]
